# Dietary supplemental plant oils reduce methanogenesis from anaerobic microbial fermentation in the rumen

**DOI:** 10.1038/s41598-020-58401-z

**Published:** 2020-01-31

**Authors:** Julio Ernesto Vargas, Sonia Andrés, Lorena López-Ferreras, Timothy J. Snelling, David R. Yáñez-Ruíz, Carlos García-Estrada, Secundino López

**Affiliations:** 1 Instituto de Ganadería de Montaña (CSIC-Universidad de León), Departamento de Producción Animal, Universidad de León, E-24007 León, Spain; 2 Universidad de Caldas, Facultad de Ciencias Agropecuarias, Grupo CIENVET, Manizales, Colombia; 3 Department of Physiology/Metabolic Physiology, Institute of Neuroscience and Physiology, The Sahlgrenska Academy, University of Gothenburg, Medicinaregatan 11, PO Box 434, SE-405 30 Gothenburg, Sweden; 4 Animal Production, Welfare and Veterinary Sciences, Harper Adams University, Edgmond, Shropshire TF10 8NB UK; 5 The Rowett Institute, University of Aberdeen, Foresterhill, Aberdeen, AB25 2ZD UK; 6 Estación Experimental del Zaidín, CSIC, 18008 Granada, Spain; 7 INBIOTEC, Instituto de Biotecnología de León, Avda. Real no. 1, Parque Científico de León, 24006 León, Spain

**Keywords:** Microbial ecology, Microbiome, Environmental impact

## Abstract

Ruminants contribute to the emissions of greenhouse gases, in particular methane, due to the microbial anaerobic fermentation of feed in the rumen. The rumen simulation technique was used to investigate the effects of the addition of different supplemental plant oils to a high concentrate diet on ruminal fermentation and microbial community composition. The control (CTR) diet was a high-concentrate total mixed ration with no supplemental oil. The other experimental diets were supplemented with olive (OLV), sunflower (SFL) or linseed (LNS) oils at 6%. Rumen digesta was used to inoculate the fermenters, and four fermentation units were used per treatment. Fermentation end-products, extent of feed degradation and composition of the microbial community (qPCR) in digesta were determined. Compared with the CTR diet, the addition of plant oils had no significant (*P* > 0.05) effect on ruminal pH, substrate degradation, total volatile fatty acids or microbial protein synthesis. Gas production from the fermentation of starch or cellulose were decreased by oil supplementation. Methane production was reduced by 21–28% (*P* < 0.001), propionate production was increased (*P* < 0.01), and butyrate and ammonia outputs and the acetate to propionate ratio were decreased (*P* < 0.001) with oil-supplemented diets. Addition of 6% OLV and LNS reduced (*P* < 0.05) copy numbers of total bacteria relative to the control. In conclusion, the supplementation of ruminant diets with plant oils, in particular from sunflower or linseed, causes some favorable effects on the fermentation processes. The addition of vegetable oils to ruminant mixed rations will reduce methane production increasing the formation of propionic acid without affecting the digestion of feed in the rumen. Adding vegetable fats to ruminant diets seems to be a suitable approach to decrease methane emissions, a relevant cleaner effect that may contribute to alleviate the environmental impact of ruminant production.

## Introduction

Ruminant herbivory is characterized by foregut microbial anaerobic fermentation of structural carbohydrates from fibrous plant-based feedstuffs providing nutrients for the animal^1^. Moreover, ruminal microbes break down all components of the diet, with characteristic pathways involved in the degradation of fiber and non-structural carbohydrates, proteins and lipids^2,3^.

Ruminal fermentation can be considered beneficial in many aspects, but has also impacts on the environment. One of the global environmental impacts of livestock agriculture is the release of greenhouse gases (GHG) into the atmosphere^4,5^, in particular methane, a gas responsible for 20% of the global warming produced by all GHG^6,7^. It is estimated that raising farm animals accounts for about 16% of anthropogenic GHG emissions, with two-thirds of the total livestock GHG emissions produced by ruminants. Methane (CH_4_) is produced in the rumen by the methanogens, archaea microbes that are closely associated with bacterial and ciliate protozoal H_2_-producing species^8,9^. The amount of methane emitted is strongly related to feed intake and diet composition. Thus, reductions in GHG from ruminants of up to 60% can be achieved just by dietary intervention, with a number of nutritional strategies proposed to reduce methane emissions^10–12^. One of these strategies is the supplementation of feed with plant oils^13–15^.

High-concentrate total mixed rations are provided to ruminants to achieve increased production, and it is becoming common practice to add fats as a concentrate source into ruminant diets to increase the energy density of the ration. Given the multiple and complex interactions between rumen microbes and diet^1^, dietary oil supplements may cause changes in the microbial community and thus fermentation processes in the rumen. The inclusion of fats in ruminant diets is considered a promising approach to manipulate the rumen microbial community to reduce methane emissions^9,16–18^. Reduction of methane production is a result of direct effects on the growth of these microbes^19^ or the metabolism pathways involved in methanogenesis^12,20^.

However, adding fats or oils to the ration ruminant diets can have other effects on rumen microbiota and fermentation. Oil supplements can affect the microbial community by a toxic effect as is the case for species of Gram-positive bacteria and ciliate protozoa^21,22^, or limit the microbial colonization of feed particles and the access of microbial enzymes to the substrates^23,24^. Consequently, feed digestion (ruminal and total tract) may be adversely affected by the addition of lipids^23,25–28^.

There are some studies on the effects of dietary plant oil supplementation on specific rumen parameters; but there is a paucity of work considering both the effects on the metabolic products and the likely microbial mechanisms that drive them, integrating these phenomena as a whole^1,29^. Most of these studies have focused on the dietary inclusion of oils rich in polyunsaturated fatty acids (mostly soybean, linseed, fish or algal oils)^16–18^. However, only a few compare the effects of oils with contrasting fatty acid composition (differing in the proportions of oleic, linoleic and α-linolenic acids) on methane production and feed digestion in the rumen. Therefore, the objective of the study reported here was to examine the effects of vegetable oils with different fatty acid composition on rumen fermentation end-products and key members of the rumen microbiota. Special attention will be paid to the effects on methane production, to assess the potential of dietary supplementation with plant oils to mitigate environmental pollution by biogas derived from enteric fermentation in the rumen. An *in vitro* rumen simulation technique (RUSITEC^30^) was used for a precise control of the fermentation conditions and the simultaneous measurement^31,32^ of digestibility, fermentation end-products, methane production, microbial protein synthesis, and key microbial groups.

## Results

The composition of the four experimental diets is shown in Table 1. The control diet (CTR) was a high-concentrate (forage was less than 30%, with 231 g neutral detergent fiber/kg dry matter) total mixed ration formulated for high-yielding dairy ewes during lactation (153 g crude protein and 519 g non-structural carbohydrates/kg dry matter). In the supplemented diets, 6 g of olive (OLV), sunflower (SFL) or linseed (LNS) oil were added to 100 g of the control diet. There were no significant differences among diets (*P* > 0.05) in ruminal pH. Compared with the CTR, the addition of oils had no apparent effect on substrate disappearance from the nylon bags containing the incubated diet. Organic matter, neutral detergent fiber and protein digestibility were higher with LNS than with SFL diet. Methane (total daily output in mL/day, or expressed relative to total gas of fermentation or per gram of fermentable organic matter) was quantitatively reduced (*P* < 0.001) when oil supplements were added to the CTR diet (Table 2). There were no significant differences (*P* > 0.05) between the type of oil in the extent to which methane production was reduced. Total volatile fatty acid (VFA) production was not affected (*P* > 0.05) by the addition of oils to the diet, but significant effects of oil supplementation on the production of specific VFA were noticed. Thus, the production of butyrate was decreased (*P* = 0.005) when any oil was added to the diet (Table 2) and propionate and isoacids were significantly increased (*P* < 0.05) in LNS compared with the CTR diet. As a result, the acetate to propionate ratio in rumen digesta was significantly (*P* < 0.001) reduced in response to the addition of all vegetable oil treatments (Table 2), with no differences (*P* > 0.05) in this ratio among the three supplemental oils. Although ammonia production was significantly (*P* < 0.05) reduced when an oil was added to the diet, microbial protein synthesis appeared to be unaffected (Table 2).

**Table 1:** Ingredients and chemical composition of control and oil supplemented diets.

Treatment	Control	Olive Oil	Sunflower Oil	Linseed Oil
**Ingredients (g/kg dry matter)**				
Cracked corn grain	250	238	238	238
Barley grain	150	143	143	143
Soybean meal	200	190	190	190
Lucerne hay	200	190	190	190
Beet pulp	90	86	86	86
Molasses	60	57	57	57
Sodium bicarbonate	15	14	14	14
Mineral vitamin premix	35	33	33	33
Olive oil	—	60	—	—
Sunflower oil	—	—	60	—
Linseed oil	—	—	—	60
**Composition (g/kg dry matter)**				
Organic matter	925	927	930	929
Crude protein	153	145	145	147
Neutral detergent fiber	231	220	222	219
Acid detergent fiber	115	109	110	108
Ether extract	22	70	73	71
**Dietary fatty acid profile (as % of total fatty acids)**				
C14:0	0.10	—	0.02	—
C16:0	22.1	15.3	12.3	11.1
Total C18	76.9	82.1	85.2	87.4
**Dietary C18 fatty acids (as % of total C18 fatty acids)**				
C18:0	5.1	5.1	5.7	5.1
C18:1 *c*9	35.1	71.0	38.7	25.3
C18:2 *c*9*c*12	52.8	20.7	53.2	30.1
C18:3 *c*9*c*12*c*15	7.0	3.3	2.2	39.5

Fatty acid profile of oils (as % of total fatty acids).

Olive oil: 12.7% C16:0. 4.2% C18:0. 71.2% C18:1 *c*9. 7.0% C18:2 *c*9*c*12. 1.6% C18:3 *c*9*c*12*c*15.

Sunflower oil: 8.5% C16:0. 4.9% C18:0. 35.4% C18:1 *c*9. 47.8% C18:2 *c*9*c*12. 0.3% C18:3 *c*9*c*12*c*15.

Linseed oil: 6.9% C16:0. 4.6% C18:0. 19.7% C18:1 *c*9. 19.6% C18:2 *c*9*c*12. 47.5% C18:3 *c*9*c*12*c*15.

**Table 2:** Effects of oils added to the diet on ruminal fermentation in RUSITEC fermenters.

Item	Control (CTR)	Olive oil (OLV)	Sunflower oil (SFL)	Linseed oil (LNS)	SEM (n = 4)	*P* value	*P* value contrast CTR vs OIL
Effluent, mL/d	577	565	513	569	10.1	0.164	0.255
pH	6.73	6.72	6.66	6.67	0.012	0.126	0.126
**Disappearance coefficients, g digested/100 g incubated**							
Organic matter	67.4^ab^	70.8^ab^	66.1^b^	72.1^a^	1.27	0.022	0.172
Neutral detergent fiber	32.9^ab^	34.7^a^	29.0^b^	35.4^a^	1.31	0.044	0.961
Acid detergent fiber	23.4	24.3	19.7	26.9	1.58	0.082	0.879
Crude protein	49.5^ab^	54.9^a^	47.6^b^	54.7^a^	1.83	0.036	0.197
**Fermentation gas production**							
Total gas, L/d	3.01	2.75	2.81	2.98	0.066	0.470	<0.001
Methane, mL/100 mL total gas	7.04^a^	5.79^b^	5.66^b^	5.57^b^	0.108	0.002	<0.001
Methane, mL/d	212^a^	160^b^	162^b^	166^b^	12.5	0.040	0.006
Methane, mmol/g fermentable OM	1.02^a^	0.81^b^	0.78^b^	0.73^b^	0.045	0.005	<0.001
**Volatile fatty acid (VFA, mmol/d)**							
Acetate	24.9	23.8	22.0	24.1	0.455	0.206	0.160
Propionate	8.39^b^	10.30^ab^	9.87^ab^	11.88^a^	0.342	0.003	0.002
Butyrate	7.38^a^	5.64^b^	5.50^b^	5.51^b^	0.166	0.005	<0.001
Isoacids	2.03^b^	2.20^ab^	2.13^ab^	2.52^a^	0.055	0.045	0.070
Total VFA	51.2	48.5	46.2	50.7	0.715	0.109	0.125
Acetate:propionate ratio	2.99^a^	2.32^b^	2.27^b^	2.03^b^	0.046	<0.001	<0.001
Ammonia N, mg/d	89.1^a^	72.4^b^	65.0^b^	65.0^b^	1.58	0.001	<0.001
Microbial protein, g/d	1.06	1.02	0.90	1.14	0.046	0.353	0.714
Microbial protein, g/100 g fermentable OM	11.4	9.2	9.6	11.2	0.72	0.158	0.127

CTR = Control diet; OLV = control diet supplemented with 6% olive oil; SFL = control diet supplemented with 6% sunflower oil; LNS = control diet supplemented with 6% linseed oil.

SEM = standard error of the mean; OM = organic matter.

Contrast CTR vs OIL: comparison between CTR and all treatments supplemented with oil.

^a,b^Within a row, mean values without common superscript letters differ (*P* < 0.05).

Fermentation kinetics (gas production after 24 h incubation, rate of fermentation) were reduced when either starch or cellulose were incubated in LNS compared to CTR inocula (Table 3). In general, the gas production derived from the fermentation of starch or cellulose was significantly reduced when the substrates were incubated with ruminal inocula from fermenters fed with oil-supplemented diets (Table 3). Fractional fermentation rate of starch and gas production after 24 h of incubation of starch were decreased (*P* ≤ 0.001) by the three supplemental oils. Similarly, the volume of gas production at 24 h of incubation of cellulose and the average fermentation rate of cellulose in batch cultures were decreased (*P* ≤ 0.001) when liquid contents from RUSITEC vessels supplemented with an oil were used as inoculum.

**Table 3:** Effects of oils added to the diet RUSITEC fermenters on fermentation kinetics of starch and cellulose in batch cultures.

Item	Control (CTR)	Olive oil (OLV)	Sunflower oil (SFL)	Linseed oil (LNS)	SEM (n = 4)	*P* value	*P* value contrast CTR vs OIL
**Starch**							
Asymptotic gas production, mL/g	311	310	301	306	17.6	0.947	0.742
Fractional fermentation rate, h^−1^	0.059^a^	0.043^b^	0.039^b^	0.039^b^	0.0036	0.001	<0.001
Lag time, h	4.6^a^	2.1^b^	2.2^b^	2.4^b^	0.44	<0.001	<0.001
Gas production at 24 h, mL/g	210^a^	191^b^	171^c^	172^c^	6.1	<0.001	<0.001
Average fermentation rate, mL/h	9.44^a^	8.62^ab^	7.48^ab^	7.50^b^	0.350	0.037	0.091
**Cellulose**							
Asymptotic gas production, mL/g	352^a^	300^ab^	278^b^	271^b^	19.1	0.005	0.001
Fractional fermentation rate, h^−1^	0.018	0.017	0.016	0.014	0.0017	0.192	0.111
Lag time, h	9.8	13.7	16.7	15.2	2.64	0.112	0.030
Gas production at 24 h, mL/g	108^a^	68^b^	52^b^	49^b^	7.9	<0.001	<0.001
Average fermentation rate, mL/h	3.69^a^	2.70^b^	2.34^b^	2.11^b^	0.195	<0.001	<0.001

CTR = Control diet; OLV = control diet supplemented with 6% olive oil; SFL = control diet supplemented with 6% sunflower oil; LNS = control diet supplemented with 6% linseed oil.

SEM = standard error of the mean.

Contrast CTR vs OIL: comparison between CTR and all treatments supplemented with oil.

^a,b^Within a row, mean values without common superscript letters differ (*P* < 0.05).

**Figure 1:**
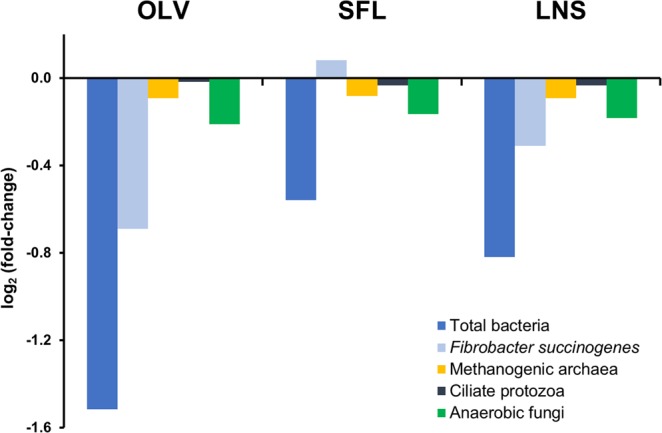
Relative quantitation compared to control diet of copy numbers of rumen microbial groups (major prokaryotic and eukaryotic domains including the methanogen *mcrA* involved in methanogenesis) after supplementation with olive (OLV), sunflower (SFL) or linseed (LNS) oil. Fold-changes for specific amplicon groups were calculated as the (log 2) ratio of normalized copy numbers.

Relative to the control diet, copy number of total bacteria (Fig. 1) was significantly reduced by the addition of either OLV (*P* < 0.001) or LNS oil (*P* = 0.044). The same inclusion level of SFL led to a non-significant (*P* = 0.095) reduction. The addition of oils did not affect significantly the copy number of ciliate protozoa (*P* = 0.98), fungi (*P* = 0.74) or methanogen *mcrA* (*P* = 0.66) in RUSITEC digesta (Fig. 1). There were no significant (*P* = 0.61) differences among control and oil-supplemented diets in the copy number of *Fibrobacter succinogenes*, one of the main fibrolytic bacteria species.

## Discussion

The study reported herein used a tested *in vitro* technology to simulate the effects of supplementing a high-concentrate diet with different vegetable oils, which varied in fatty acid composition, on ruminal fermentation and microbial community composition. The study was designed to measure diverse aspects of rumen metabolism using simultaneous measurements, including nutrient digestion, methane production and changes in the microbiota in the digesta. A number of previous studies have reporting results on the effects of lipid supplementation on some parameters of rumen fermentation, however, there are very few that address all these aspects joint- and simultaneously. Obtaining *in vivo* digesta samples can be challenging and require invasive procedures or surgery. However, the rumen simulation technique (RUSITEC) is an effective tool to characterize ruminal fermentation *in vitro* and allows precise control of culture conditions and of the inputs and outputs in each fermentation unit. *In vitro* systems for continuous culture of mixed rumen microorganisms contribute to reduce the use of experimental animals, and have been used previously to investigate effects of lipid supplementation^31^.

Supplementary fat increases the energy concentration of the diet but may also affect microbial fermentation and feed degradation in the rumen^27^. Supplementary lipids are associated with inhibition of the rumen microbiota or a physical coating of feed particles limiting their comminution and degradation^21^. However, nutrient degradation (organic matter or fiber) was not apparently affected when measured as substrate disappearance from feed containers (artificial fiber bags) in the RUSITEC fermenters. Furthermore, other parameters related to the extent of feed degradation in the rumen, such as fermentation gas or VFA production, were not affected by the addition of oils to the diet, indicating that effects of oil supplementation at 6% on feed digestibility would be of little biological relevance. The effects of lipids on rumen fermentation become more evident when purified substrates are fermented^24^, and affect mainly fiber degradation in the rumen^23^, with minor effects on the digestion of non-structural carbohydrates or protein^26^. It is worth mentioning that the amount and type of lipid added^23,33^ and the diet composition^33,34^ will determine the magnitude of the effects of dietary lipid supplementation on nutrient degradation. Our results are comparable to those reported by other authors showing minor effects of supplemental fats on extent of ruminal fermentation^35,36^. Doreau *et al*.^37^ found that the dietary addition of oils at levels similar to that used in our study did not affect organic matter fermentation in the rumen. In a pooled analysis of a number of published studies, Hess *et al*.^38^ concluded that fats can be added to high-concentrate diets up to 6% with no effects on feed digestibility. Other meta-analysis showed that total tract and ruminal fiber digestibility were not affected by the supplementation of dairy cow diets with fats^28^. Patra^39^ concluded that fat had to be added to sheep diets at high concentrations to affect fiber digestibility adversely based on the available information, and Knapp *et al*.^14^ recommended levels of addition of fats to total mixed rations of up to 6–7%.

Plant oils added to feed can also cause changes in the ruminal microbiota. In our study, total viable bacteria numbers were significantly reduced. Rumen bacteria can be hindered by dietary fats and oils as shown in studies *in vivo*^40,41^ and in pure cultures *in vitro*^42^, with results supporting a dose-related effect. Nur Atikah *et al*.^43^ did not observe changes in *Fibrobacter succinogenes* or *Ruminococcus* spp. numbers following addition (6%) of either olive or sunflower oil to high-concentrate diets fed to goats. Similar results (lack of effects on rumen microbiota) were reported *in vitro* with 3% soybean oil supplementation in RUSITEC^44,45^. Other microbial groups that are involved in fiber degradation (ciliate protozoa and fungi) were not consistently affected by the vegetable oils. Changes in the bacterial community were reflected in the fermentation end-products profile. Thus, total VFA and acetate were not affected, but propionate increased leading to reduced acetate to propionate ratio in agreement with previous reports^46–48^. Decrease in butyrate output in response to supplementation of all oil types was in agreement with other studies^46,49^ and most likely due to the high sensitivity of butyrate-producing bacteria to polyunsaturated fatty acids^22^. Finally, the supplemental oils reduced the daily ammonia output with no effects on microbial protein synthesis. Similar effects have been reported by other authors^16,50^. The effects of fats on protein degradation and synthesis of microbial protein in the rumen may be affected by the type of diet fermented and by the persistence of ciliate protozoa in the RUSITEC fermenters^51,52^. The reduction in ammonia output in the rumen by the addition of dietary oils could be environmentally beneficial decreasing the emissions of N from ruminants. These emissions contribute to environmental pollution mainly through the leaching to surface and ground water resources^53,54^. There are, however, contrasting results showing that with some oils the beneficial effect decreasing methane may be traded-off with an increased N excretion^55,56^.

Daily methane produced (per unit of fermented OM) from ruminal fermentation was decreased by more than 20% when the basal diet was supplemented with any of the plant oils examined. The depressing effect of supplemental plant oils on methanogenesis cannot be attributed to a general inhibitory effect on ruminal fermentation, as the indicators of the extent of degradation (dietary OM rumen digestibility, production of fermentation gas or daily VFA output) were not affected by any of the supplemental plant oils^7^. Additionally, not only the total daily amount of methane generated was reduced, but also the concentration of methane in the fermentation gas, reinforcing the idea that supplemental plant oils may exert a rather specific effect on methanogenesis. Decreasing methane production in the rumen in response to the addition of oils to the diet without affecting rumen fermentation has been reported by other authors^45,57,58^, and confirmed in meta-analyses pooling data from different studies^39,59^. In agreement with Jalč *et al*.^60^, this effect was similar regardless the fatty acid composition of the plant oil added. The dietary addition of C18 free fatty acids decreases methane production from ruminal fermentation, and the extent of this decrease is affected by the degree of fatty acid unsaturation, the inclusion level and the basal diet^57,61^. When oils or oilseeds differing in their fatty acid (mostly esterified) composition are used as dietary lipid supplements, most of them decrease methane production, with minor differences among the diverse oils or oilseeds^49,62–64^. Differences among supplemental oils in their effect on ruminal methane production are highly dependent on the basal diet, and it seems that the extent of the decrease in methane caused by the addition of different oils is similar when added to a high-concentrate diet^33,63^, as that used in our study. Although the daily output of VFA was not affected by oils, a shift in the fermentation pattern became apparent with a decreased acetate to propionate ratio, in agreement with other authors^44,45^. With the formation of more propionate less H_2_ would be available for methanogenesis^65^. The inhibitory effects of fats on rumen methanogenesis have been explained also considering the changes in the rumen microbiota, as more dietary oils have been associated to a reduction of ciliate protozoa and methanogen archaea in the rumen^9,17,18,66^. Methanogenic archaea use the available hydrogen in the rumen for methane production^1^. However, Nur Atikah *et al*.^43^ observed increased methanogen numbers in response to the addition of OLV or SFL oils. In our study, the relative numbers of protozoa and methanogen *mcrA* were not affected by oil supplementation. However, ciliate protozoa numbers are not sustainable in RUSITEC fermenters^67^. As for methanogenic archaea, it is noteworthy that the copy number of the functional gene *mcrA* involved in the methanogenesis was quantified by PCR. The quantification of particular groups of methanogens of interest by specific primers targeting specific 16S rRNA genes would have provided some valuable additional information^68–70^. Biohydrogenation of unsaturated fatty acids can compete with methanogenesis for metabolic H_2_^18^. It can be expected that considerable amounts of H_2_ will be used for the saturation of fatty acids provided with the oil supplemented diets^71^, and thus diverted from methanogenesis.

In conclusion, the supplementation of ruminant diets with plant oils, in particular from sunflower or linseed, causes some favorable effects on ruminal fermentation. Methane production in the rumen is reduced by adding vegetable oils to high concentrate mixed rations. Concomitantly, ruminal fermentation is altered with a shift towards more propionate production, without affecting the extent of digestion of feed. No substantial differences are observed among vegetable oils (olive, sunflower or linseed) differing in their polyunsaturated fatty acid composition. The supplemental oils seem to decrease also the output of ammonia from protein degradation, although it seems necessary to evaluate how dietary strategies influence both N excretion and methane emissions in ruminants. Considering the observed reduction in methane production, it is possible to propose the addition of plant oils to ruminant rations as a feasible and relatively inexpensive nutritional strategy with a cleaner repercussion on the environment. In this way, the addition of vegetable oils lipids to ruminant diets can improve feed efficiency and attenuate the environmental impact of ruminal fermentation contributing to more efficient, sustainable and cleaner animal production systems, although these effects need to be confirmed under field *in vivo* conditions.

## Methods

### Experimental design

Ruminal fermentation was simulated *in vitro* using semi-continuous flow fermenters (RUSITEC) for the culture of mixed ruminal microorganisms^30^. The study was designed to investigate the effects of supplementing a high concentrate diet with vegetable oils differing in their fatty acid composition, for a total of four experimental treatments (diets). The control diet (CTR) was a standard high-concentrate total mixed ration for ewes during lactation, and had no supplemental oil (Table 1). Three vegetable oil treatments were prepared by supplementing the control diet with either olive oil (OLV), sunflower oil (SFL) or linseed oil (LNS) in all cases at a concentration of 60 g/kg (6%) (Table 1). Two RUSITEC systems, each with eight fermentation units, were used having four fermenters (two in each RUSITEC system) for each experimental diet. The experiment followed a randomized complete block design, with four experimental treatments (diets) allocated in two blocks (RUSITEC systems). The experimental unit was each fermentation vessel, resulting in four replicates per treatment.

### Procedures, sampling and measurements

Rumen digesta obtained from four sheep fed a diet with 70% grass hay and 30% concentrate was used to inoculate the RUSITEC fermenters^45^. Sheep care and handling were in accordance with the principles of the EU Directive 2010/63 on the protection of animals used for specific purposes. The Animal Ethics Committees of CSIC and University of Leon (Spain) had approved the procedures for digesta sampling from sheep. Each fermenter was operated daily as described in detail by García-González *et al*.^72^ and Vargas *et al*.^45^. Artificial saliva (pH 8.4, 550 mL/day) was continuously infused to each vessel and incubation temperature was set at 39 °C. Steady state was achieved after 6-days and thus measurements and samplings were initiated afterwards. Feed (15 g of one of the experimental diets) was provided inside nylon bags (100 µm pore size) placed into a holed plastic container introduced into each fermentation vessel. Bags were withdrawn after 48 h of incubation and replaced with others providing feed. The undigested residue remaining in the bags after 48 h was used to calculate the diet digestibility (as matter disappearance from the bag) in the rumen. The liquid flowing out the vessel (effluent) was collected in conical flasks, and a sample (50 mL) was collected daily and frozen (−18 °C) for ammonia and VFA analysis. Fermentation gas was collected in airtight bags, volume of gas produced daily was measured using a flow meter, and samples of gas were taken in hermetic vacuum glass tubes. All these samples (incubation residues, effluent and gas) were collected on days 7, 8, 9, 14, 15 and 16 of the experiment, and the values recorded were averaged across the six measurement days for each vessel.

A representative sample of total digesta (effluent + bag residue) was collected on day 12 after infusing (^15^NH_4_)SO_4_ into the vessels for three days^45^. A microbial pellet was isolated from this sample and microbial protein output from each vessel was determined using ^15^N as a marker as described by Carro and Miller^73^. On day 17, samples of digesta were obtained by mixing liquid content with bag residues from each vessel, frozen at −80 °C and freeze-dried. DNA was extracted from these latter samples and then used for quantitative real-time polymerase chain reaction (qPCR) to quantitate the abundance of some key microbial groups^45^.

Fermentation kinetics of pure substrates (starch or cellulose) were determined using the *in vitro* gas production technique^74^, and the procedures described in detail by Vargas *et al*.^45^. Once the RUSITEC study was finished, the liquid contents of each fermenter were used as inoculum for batch cultures incubated in 120-mL serum bottles. In each bottle 500 mg of each substrate were weighed and mixed with 50 mL of fluid from each RUSITEC vessel. The bottles were sealed and incubated at 39 °C, measuring the volume of fermentation gas released to the headspace by using a pressure transducer. Gas measurements were recorded at different time intervals from inoculation to 92 h of incubation^45^, building up a curve plotting cumulative gas production against incubation time. Fermentation kinetics (extent and rate of degradation of each substrate) were derived by fitting the exponential model of France *et al*.^75^ to each gas production curve. As for each substrate the only difference was the inoculum used for the cultures, the fermentation kinetics were assumed to characterize the amylolytic or cellulolytic activity in each RUSITEC vessel as affected by its diet (unsupplemented or supplemented with any of the plant oils).

### Chemical and qPCR analyses

Analytical methods for the determination of chemical composition (proximate analyses), VFA in effluent and methane in fermentation gas (gas chromatography), ammonia nitrogen in effluent (colorimetry), and non-ammonia N and ^15^N enrichment in digesta and microbial pellets (isotope ratio mass spectrometry) were described in detail by García-González *et al*.^72^. Fatty acids in oils and diets were determined according to Morán *et al*.^76^.

Real-time PCR was carried out using the procedures and primers detailed by Vargas *et al*.^45^ for the quantitative estimation of copy numbers of bacteria, *Fibrobacter succinogenes*, ciliate protozoa, fungi and methanogenic archaea in the digesta samples collected from each vessel at the end of the study. Standards for PCR quantitation of each microbial group were obtained from plasmids in which the specific PCR amplicon sequence had been inserted^40^.

### Statistical analysis

Data were subjected to ANOVA using the SAS software package (SAS Institute Inc. 2011. SAS/STAT® 9.3 User’s Guide. Cary, NC: SAS Institute Inc.). The statistical model included three sources of variability namely the fixed effect of diet (CTR, OLV, SFL or LNS), the blocking factor (RUSITEC system 1 or 2) and the residual error. The Tukey test was used for the multiple comparisons of means, and the effect of adding any vegetable oil to the diet was tested by an orthogonal contrast.
